# Epithelial interferon-γ responsiveness shapes chemokine induction and clinical outcomes in herpes simplex virus infection

**DOI:** 10.1371/journal.ppat.1014612

**Published:** 2026-09-30

**Authors:** Angelina Zimenko, Ian Hayman, Youyi Fong, Luke D. Monroe, Margaret G. Mills, Alex Greninger, Christine Johnston, Lawrence Corey, Jia Zhu

**Affiliations:** 1 Department of Laboratory Medicine and Pathology, University of Washington School of Medicine, Seattle, Washington, United States of America; 2 Vaccine and Infectious Disease Division, Fred Hutchinson Cancer Center, Seattle, Washington, United States of America; 3 Department of Medicine, University of Washington School of Medicine, Seattle, Washington, United States of America; 4 Institute of Stem Cell and Regenerative Medicine, University of Washington, Seattle, Washington, United States of America; Cardiff University, UNITED KINGDOM OF GREAT BRITAIN AND NORTHERN IRELAND

## Abstract

Herpes simplex virus type 2 (HSV-2) infects genital epithelia and causes a wide spectrum of clinical outcomes, ranging from asymptomatic viral shedding to recurrent self-limited genital lesions. Interferon gamma (IFNγ) is critical in tissue-resident T-cell mediated antiviral defense against HSV-2 reactivation. However, the responsiveness of epithelial cells to IFNγ and its contribution to disease variability remains poorly understood. Here we investigated IFNγ-mediated antiviral responses in primary keratinocytes and donor-matched fibroblasts from individuals with either asymptomatic or ulcerative HSV-2 infection. Our results revealed that keratinocytes are remarkably sensitive to IFNγ, with altered gene expression observed at doses much lower than previously reported. Keratinocytes exhibited greater sensitivity to IFNγ stimulation than fibroblasts, including robust induction of chemokines involved in immune cell recruitment. Among them, CXCL10 was the most sensitive and strongly induced gene, displaying rapid upregulation even at low IFNγ concentrations. Notably, keratinocytes from individuals with asymptomatic HSV-2 infection exhibited significantly greater IFNγ responses at both the mRNA and protein levels compared with those from symptomatic donors, particularly at physiologically low IFNγ doses. This heightened responsiveness was associated with increased nuclear localization of phosphorylated STAT1 following IFNγ stimulation, despite similar baseline expression of IFNγ receptor and STAT1 across donor groups. While IFNγ pretreatment similarly inhibited HSV replication in keratinocytes from both cohorts, the enhanced chemokine production by keratinocytes from asymptomatic individuals may promote more efficient recruitment of antiviral immune cells. Together, these findings identify intrinsic variation in epithelial IFNγ signaling as a potential host factor associated with clinical outcomes in HSV infection. The results suggest that CXCL10-mediated immune recruitment might be a key mechanism linking tissue-resident T cell cytokine signaling to epithelial antiviral defense. Understanding variability in epithelial barrier immune defense may be crucial to improving therapies and vaccine strategies for genital herpes infection.

## Author summary

Genital herpes caused by herpes simplex virus type 2 (HSV-2) affects millions worldwide, yet some individuals experience frequent painful outbreaks while others remain symptom-free. We investigated whether skin epidermal keratinocytes, the primary cell type involved in HSV-2 infection initiation, respond differently to immune signals in people with asymptomatic versus recurrent ulcerative genital herpes. We revealed that keratinocytes from individuals with asymptomatic infection were more sensitive to interferon gamma (IFNγ), a key antiviral molecule, and produced higher levels of CXCL10, a crucial protein for recruiting protective immune cells. In contrast, keratinocytes from individuals with ulcerative disease demonstrated weaker responses to IFNγ signaling. These findings suggest that epithelial sensitivity to IFNγ is an important determinant of genital herpes disease severity and could guide new strategies to improve immune control of HSV-2.

## Introduction

Herpes simplex virus (HSV) establishes lifelong infection in humans, characterized by periods of latent infection in neurons and episodic reactivation with ulceration in the orolabial mucosa and genital tract. Globally, HSV infection is highly prevalent, with HSV type 1 (HSV-1) infecting an estimated 5.3 billion individuals and HSV type 2 (HSV-2) affecting over 500 million individuals [1–3]. Genital infection with either HSV-1 or HSV-2 can lead to severe consequences, including neonatal herpes, and HSV-2 infection increases the risk of acquiring and transmitting human immunodeficiency virus (HIV) [1,2,4–6]. HSV reactivation is triggered by various physiological stimuli initiating viral gene expression and production of new virions in latently infected neurons [7,8]. Upon reactivation, nascent virions travel from nerve endings to infect the epithelial cells in the genital skin and mucosa [7].

Viral reactivation can manifest as ulcer-forming lesions or asymptomatic shedding, with considerable variation in frequency and duration of recurrences among individuals [4,9]. Approximately 50% of HSV-2 shedding episodes are rapidly contained within 6–18 hours [10] while others can persist for more than a week, resulting in a spectrum of clinical diseases [4,9,10]. This variability in episode duration and severity suggests that differences in immune responses at the individual level could contribute to the wide range of clinical disease expression. A better understanding of the molecular mechanisms underlying effective immune surveillance and rapid control is essential for improved interventions for genital herpes, for which there is currently neither a cure nor a vaccine.

Tissue-resident memory CD8^+^ T cells (CD8 TRMs) are critical for clearing and containing HSV reactivation in humans and animal models [11–15]. These immune cells persist at sites of prior infection, acting as sentinels with effector functions that surpass those of circulating T cells. CD8 TRMs eliminate invading pathogens by releasing cytolytic granules and antiviral cytokines and chemokines [15–19]. Their ability to confer tissue-wide protection despite limited numbers suggests that cytokine-mediated responses are essential for controlling HSV dissemination [20–23].

Interferon gamma (IFNγ), a Type II interferon, is a key cytokine involved in antiviral defense [24–27]. IFNγ treatment can condition keratinocytes, the primary targets of HSV infection, to restrict viral replication and produce chemotactic signals that recruit and sustain antiviral immune responses [20,21,23,27,28]. Stimulation with IFNγ leads to broad transcriptional changes in keratinocytes, altering the expression of hundreds of genes within 24 hours. Moreover, elevated IFNγ levels can be detected at sites of prior HSV lesions weeks after healing [20]. Notably, peripheral blood mononuclear cells (PBMCs) from asymptomatic donors produce significantly more IFNγ in response to HSV antigens than those from individuals with recurrent ulcerative disease [29].

Individuals with asymptomatic HSV reactivation may produce more IFNγ, but it is not known whether the targets of IFNγ signaling, including structural cells such as keratinocytes, are more sensitive to IFNγ than keratinocytes from individuals with ulcerative HSV disease. We hypothesized that keratinocyte sensitivity to IFNγ is associated with disease severity, with keratinocytes from asymptomatic donors exhibiting stronger or more rapid responses to low IFNγ doses compared to those from donors with ulcerative HSV disease. To test this hypothesis, we focused on five IFNγ stimulated genes, *CXCL10* [30–32]*, CCL5* [33,34], *IL12A* [35,36], *IL15* [37,38], and *IFI16* [39–41], which have established or putative roles in controlling HSV infection [20].

CXCL10 and CCL5 (also known as RANTES) are chemokines induced by IFNγ that play key roles in recruiting T cells to the site of infection. CXCL10 is rapidly induced and recruits T cells, thereby enhancing adaptive immune responses against viral infections, and has been shown to affect the efficacy of experimental HSV vaccines [32,33,42–46]. In contrast, CCL5 displays a delayed expression compared to CXCL10. The cytokines IL-12 and IL-15 are important for the retention and function of immune cells at infection sites. IL-12 amplifies IFNγ signaling by promoting further IFNγ production from nearby T cells [47–49], while IL-15 is essential for the development, maintenance, activation, and effector functions of CD8  TRMs [50–55]. IFI16 is a nuclear sensor that detects viral DNA, epigenetically silences HSV gene expression, and restricts viral replication. IFI16 has also correlated with the severity of HSV symptoms [40,41,56].

To test our hypothesis, we recruited donors with asymptomatic or ulcerative HSV-2 infection (≥6 ulcerative episodes per year). We identified distinct gene expression signatures induced by IFNγ between donor-matched keratinocytes and fibroblasts. Although much of HSV biology has historically been studied in fibroblasts, our data suggest that keratinocytes may serve as a more physiologically relevant target for studying early antiviral responses at the epithelial barrier [50]. Notably, keratinocytes from asymptomatic individuals exhibited significantly higher induction of interferon-stimulated genes (ISGs), particularly CXCL10, compared to those from individuals with ulcerative HSV. These findings indicate that structural cells contribute to donor variability in IFNγ-mediated antiviral response, which may influence disease severity, and suggest new avenues for therapeutic interventions targeting local immune control by structural cells.

## Results

### Keratinocytes exhibit donor-specific gene expression in response to IFNγ stimulation

Keratinocytes, the primary structural cells of the skin and the main targets of HSV infection, were isolated from 10 individuals, 5 asymptomatic and 5 donors with recurrent ulcerative disease (Table 1). None of the participants had any known immunocompromising conditions. Keratinocytes were treated with or without 1.0 U/mL IFNγ and harvested 4 hours post-treatment. We assessed the expression of *CXCL10, CCL5*, *IL15, IL12A,* and *IFI16* using RT-PCR. Following treatment, *CXCL10* demonstrated strong expression, accompanied by robust induction of *IL12A and IL15*. *IFI16* showed moderate induction, while *CCL5* exhibited little to no change at this early time point (Fig 1A). The magnitude of gene induction varied amongst donors (Figs 1B-F), but all donor-derived keratinocytes demonstrated increased gene expression of select genes compared to baseline levels.

**Table 1:** Subject demographics.

Donor	Subgroup	Age at Enrollment	Sex	HSV Status	Years since HSV-2 first genital episode	Number of recurrences annually	History of oral HSV	Years since first oral episode
1	Asymptomatic	63	F	HSV-1&2	Unknown	0	Yes	54.1
2	Asymptomatic	67	F	HSV-1&2	Unknown	0	Unknown	n/a
3	Asymptomatic	48	F	HSV-2	Unknown	0	No	n/a
4	Asymptomatic	56	F	HSV-1&2	Unknown	0	Yes	52.2
5	Asymptomatic	44	F	HSV-1&2	Unknown	0	Yes	34.1
6	Ulcerative	74	F	HSV-1&2	44.8	6	No	n/a
7	Ulcerative	40	F	HSV-2	2.5	8	No	n/a
8	Ulcerative	48	F	HSV-1&2	21.4	6	Yes	36.6
9	Ulcerative	41	M	HSV-1&2	3.4	8	Yes	22.5
10	Ulcerative	64	F	HSV-1&2	12.8	7	No	n/a

**Fig 1:**
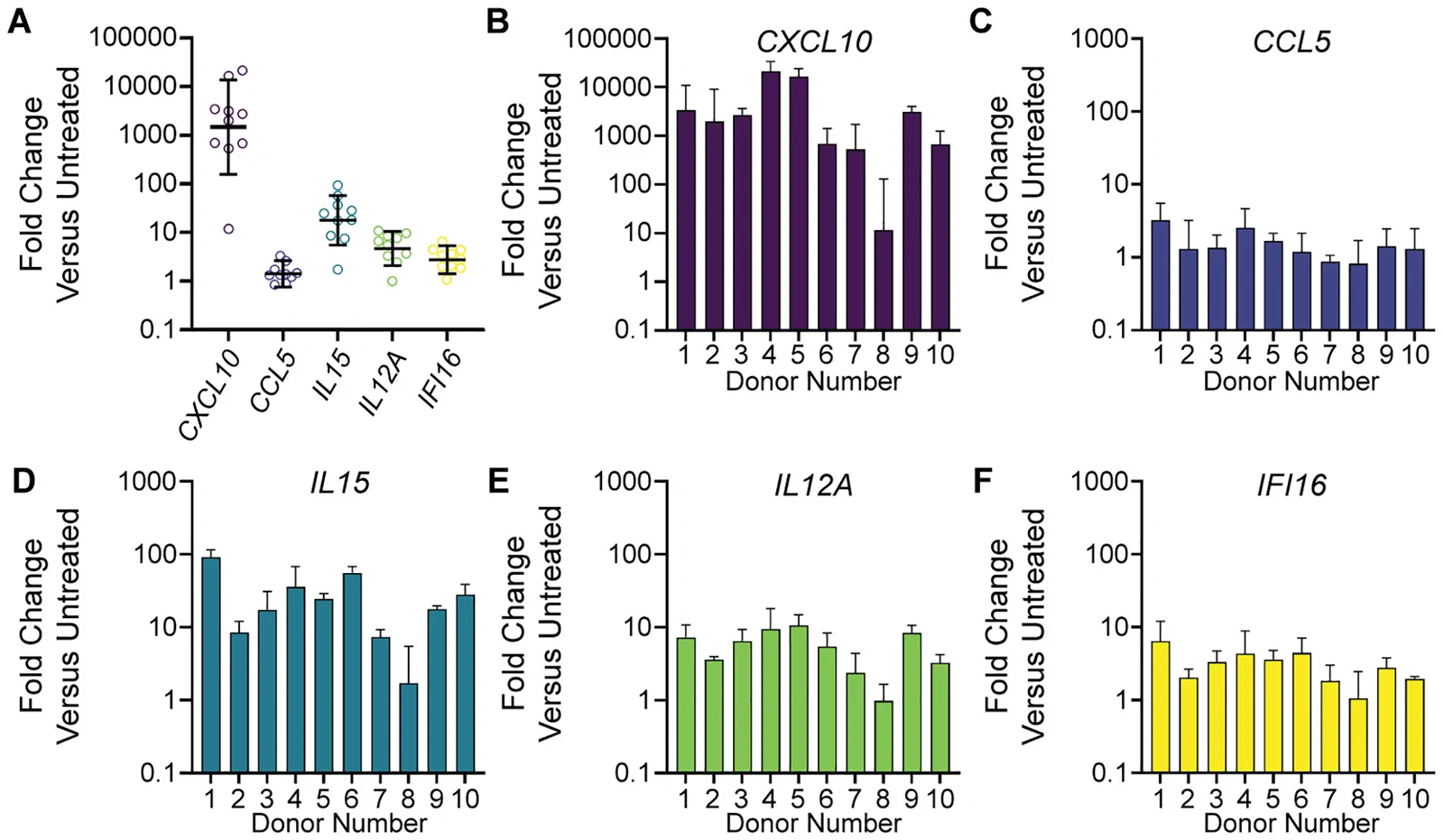
IFNγ treatment of keratinocytes and transcriptional analysis of gene expression. Donor-dependent gene induction of *CXCL10, CCL5, IL15, IL12A,* and *IFI16* in response to 1.0 U/mL IFNγ in primary keratinocytes derived from HSV-2 seropositive donors (n = 10 donors with 3 replicates per donor, A-F). Keratinocytes were treated with 1.0 U/mL IFNγ and collected 4 hours post-treatment for quantitative RT-PCR analysis **(A-F)**. Target gene expression relative to matched mock-treated cells was normalized to β-actin. **(A)** Geometric mean fold change in gene expression following IFNγ treatment. Error bars represent one standard deviation. Symbols represent the geometric mean of three replicates for each donor. **(B—F)** Geometric mean and standard deviation of log fold change gene induction for *CXCL10, IL15, IL12A, CCL5,* and *IFI16* relative to untreated cells.

CXCL10 demonstrated substantial variability between donors (an average of 1500-fold, ranged from 11.72 to 21,345 fold) (Fig 1B). In contrast, *CCL5* exhibited minimal induction (1.45-fold, 0.82 to 3.25) at 4 hours post treatment, consistent with its established role as a temporally delayed chemotactic molecule. *IL15* (Fig 1D) and *IL12A* (Fig 1E) were the second and third most induced genes, with *IL15* demonstrating an average fold-change of 18.23 (1.71 to 91.39) in expression and *IL12A* demonstrating an average fold change of 4.77 (0.98 to 10.61) in expression. *IFI16* exhibited limited induction with an average fold change of 2.81 (1.05 to 6.44) (Fig 1F) at 4 hours post treatment with IFNγ. Together, these data demonstrated remarkable donor variations in the responsiveness of primary keratinocytes to IFNγ stimulation.

### Dose-response analysis reveals extreme sensitivity of *CXCL10* expression to IFNγ in keratinocytes

To assess the sensitivity of primary keratinocytes to IFNγ, we conducted a dose-response transcriptional analysis of *CXCL10, CCL5, IL15, IL12A, and IFI16* expression. Keratinocytes were treated with increasing concentrations of IFNγ (0.1, 1.0, 10, or 100 U/mL) for 4 hours, and the relative gene expression was quantified using ΔCt values relative to β-actin (Ct[gene of interest] - Ct[β-actin]) (Fig 2A). Fold change in gene expression was calculated using the ΔΔCt method, in which we compared mock-treated controls to IFNγ-treated samples (Fig 2B).

**Fig 2:**
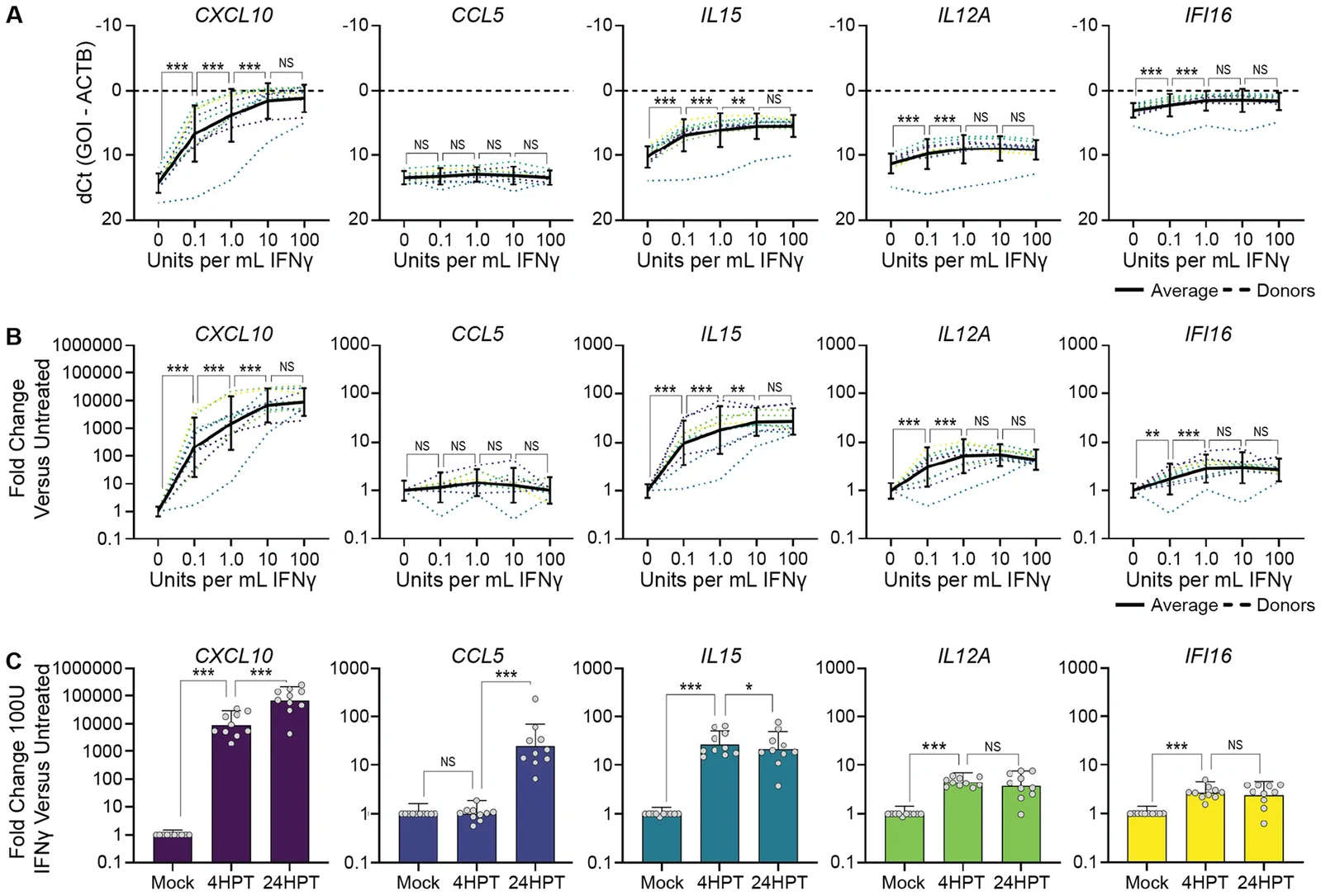
Primary keratinocytes demonstrate dose-dependent gene induction via IFNγ-treatment. **(A)** Changes of *CXCL10, CCL5, IL15, IL12A*, and *IFI16* expression in primary keratinocytes (n = 10 donors with 3 replicates per donor, A-C) 4 hours following mock-treatment or treatment with 0.1, 1.0, 10, or 100 U/mL IFNγ, normalized to β-actin (dashed line). Data is represented as ΔCt, which is calculated by subtracting the Ct value of β-actin from the Ct of induced genes. All Ct values were acquired directly from qPCR with a maximum Ct threshold of 35. **(B)** Fold change in *CXCL10, CCL5, IL15, IL12A*, and *IFI16* expression in primary keratinocytes (n = 10) 4 hours following mock-treatment or treatment with 0.1, 1.0, 10, or 100 U/mL IFNγ. Fold change is calculated as relative to mock expression, normalized to β-actin, using the ΔΔCt method. The bold line represents the average fold change of gene expression across all donors. Dashed lines represent fold change expression in individual donors. **(C)** Fold change in *CXCL10, CCL5, IL15, IL12A*, and *IFI16* expression in primary keratinocytes mock-treated or treated with 100 U/mL IFNγ at 4 hours and 24 hours post-treatment. Symbols represent the geometric mean of each individual donor. ^*^P < 0.05, ^**^ *P* < 0.01, ^***^ *P* < 0.001, NS, not significant; linear mixed models were used to analyze fold change in gene expression.

Among all genes examined, *CXCL10* displayed the lowest baseline expression (Fig 2A). Remarkably, upon IFNγ stimulation, *CXCL10* exhibited the highest fold induction across all donors (Fig 2B). At the lowest dose of IFNγ tested (0.1 U/ml), *CXCL10* expression was significantly upregulated (P < 0.001), with 7 out of 10 donors demonstrating an increase greater than 100-fold compared to untreated keratinocytes (Fig 2B). Peak *CXCL10* induction was observed at 10 U/mL IFNγ in 2 out of 10 donors and at 100 U/mL IFNγ in 8 donors (Fig 2B). In contrast, while *CCL5* exhibited baseline expression comparable to *CXCL10* (Fig 2A) *CCL5* demonstrated minimal induction and a weak dose-response to IFNγ, making it the least responsive gene across all targets (Fig 2B). *IL15* exhibited a modest dose-response to all doses of IFNγ (Fig 2A and B), with peak induction occurring at 10 U/mL IFNγ in most donors (Fig 2B). *IL12A* ranked third, with peak induction at 1.0 U/mL IFNγ in 4 donors and at 10 U/mL in 5 donors (Fig 2B). *IFI16* exhibited the highest baseline expression and showed minimal, but significant, induction following IFNγ treatment (Fig 2A and B).

To elucidate whether prolonged IFNγ stimulation alters gene expression, we compared gene induction in primary keratinocytes at 4 hours and 24 hours post treatment with IFNγ (Fig 2C). Since all donors exhibited maximum induction at 100 U/mL IFNγ across all target genes (Fig 2A and 2B), we standardized the treatment to 100 U/mL IFNγ to minimize variability in gene expression due to dose effect in this experiment. *CXCL10* expression increased substantially over the 24-hour period, with an average fold change in expression from 8,941–66,578 (*P* < 0.001), suggesting continuous induction, possibly through a positive feedback loop. Notably, *CCL5* expression was minimal at 4 hours, but was significantly induced at 24 hours (23.8-fold change, *P* < 0.001), consistent with a marked delay in expression relative to other ISGs. *IL15* expression decreased slightly from 4 to 24 hours (*P <* 0.05), whereas *IL12A* and *IFI16* expression remained unchanged under these conditions (Fig 2C).

Our findings demonstrate that the dosage and duration of IFNγ stimulation profoundly influence the kinetics of ISG expression in primary keratinocytes. *CXCL10* exhibits robust and sustained induction following treatment with IFNγ and *CCL5* displays delayed activation and weaker dose-response. These results underscore IFNγ-mediated gene-specific dynamics and temporal regulation in key structural cells and highlight that *CXCL10* expression is extremely sensitive to the presence of IFNγ and requires minimal stimulation for rapid and robust expression.

### Keratinocytes elicit greater gene induction upon IFNγ stimulation than fibroblasts

HSV primarily infects keratinocytes found in the skin epidermis. Fibroblasts, predominant in the dermis and susceptible to HSV-2 infection, are located in proximity to keratinocytes within the skin [57]. Given the physical and functional relationship of these cell types, we sought to determine whether IFNγ induces cell-type specific responses in ISG expression in keratinocytes and fibroblasts. We performed an IFNγ dose-response analysis in donor-matched primary keratinocytes and fibroblasts to examine differential expression of *CXCL10, CCL5, IL15, IL12A*, and *IFI16* at 4 hours after treatment with IFNγ.

At baseline (0.0 U/mL IFNγ), fibroblasts exhibited significantly higher expression of *CCL5* (*P* ≤ 0.01), as well as *CXCL10*, *IL12A*, and *IL15* (*P* < 0.001), compared to keratinocytes (Fig 3A). Following IFNγ stimulation, keratinocytes exhibited a much larger fold increase in *CXCL10*, *IL15, and IL12A* transcription than fibroblasts across all doses (*P* < 0.001) (Fig 3B). Normalized to β-actin, the final relative expression of *CXCL10* was elevated in keratinocytes, whereas *IL15* expression did not differ between the two cell types and *IL12A* remained greater in fibroblasts (Fig 3A). Donor-derived keratinocytes demonstrated significantly higher *CXCL10* induction across all IFNγ doses compared to their fibroblast counterparts, even at maximum IFNγ stimulation (8941-fold versus 410-fold at 100 U/mL IFNγ, *P* < 0.001) (Fig 3B). Although keratinocytes did not reach the same normalized levels of *IL12A* as fibroblasts, they exhibited greater induction of *IL12A* (4.43-fold versus 1.83-fold at 100 U/mL IFNγ, *P* < 0.001). This finding suggests that keratinocytes rely on stimulus-dependent upregulation, whereas fibroblasts display ongoing constitutive expression (Fig 3B). Despite the overall higher *CXCL10* response in keratinocytes, fibroblasts exhibited comparable dose-sensitivity patterns; peak *IL15 and IL12A* expression in fibroblasts corresponded to peak gene expression in keratinocytes at the same IFNγ doses (Fig 3B). *CCL5* and *IFI16* expression did not differ significantly between cell types, with both keratinocytes and fibroblasts exhibiting limited fold induction across the IFNγ dose range (Fig 3A and 3B).

**Fig 3:**
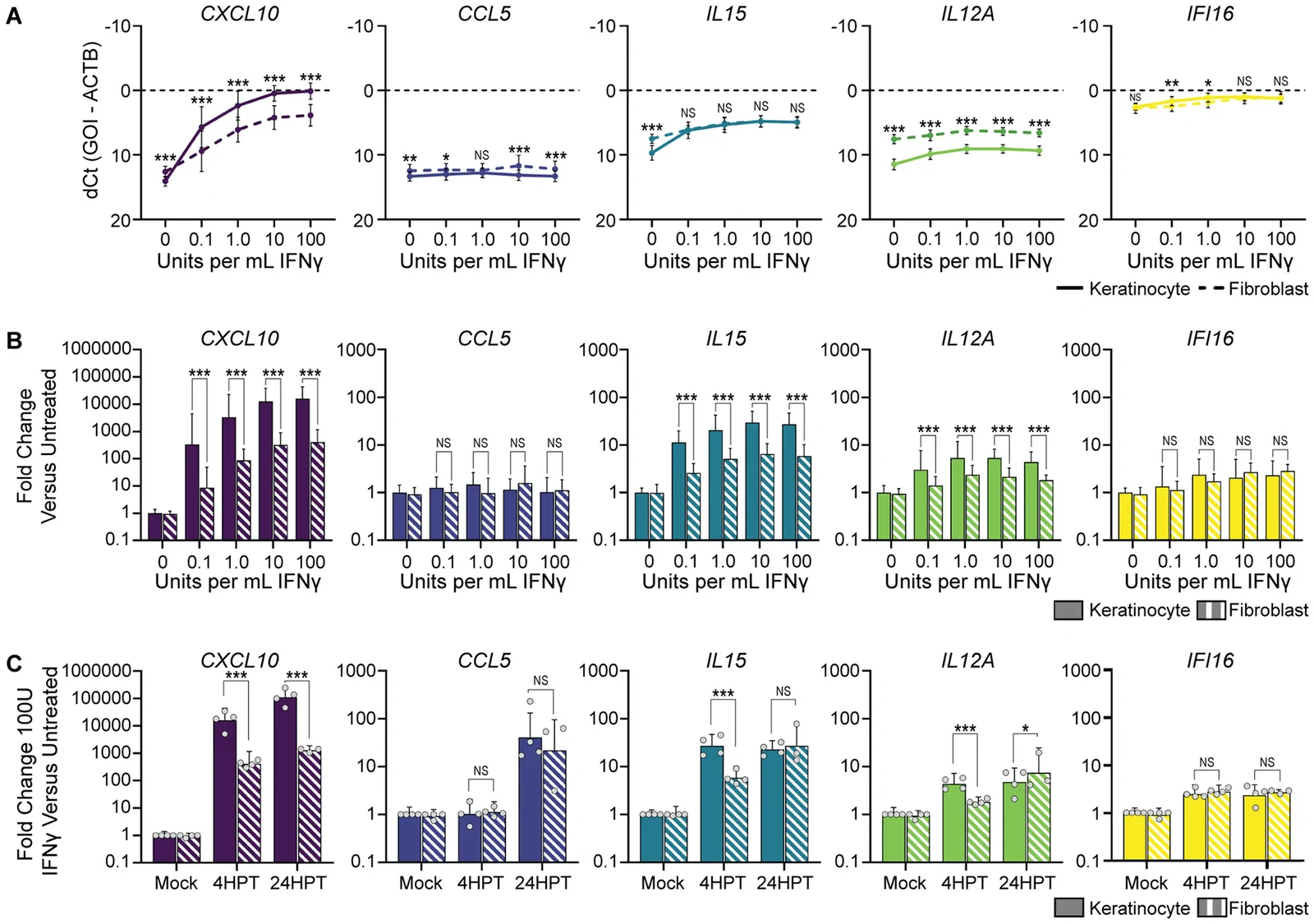
Comparison of IFNγ induced gene expression in donor-derived keratinocytes and matched donor-derived fibroblasts. **(A)** Induction of *CXCL10, CCL5, IL15, IL12A*, and *IFI16* expression in primary donor keratinocytes and donor-matched fibroblasts (n = 4 donors with 3 replicates per donor, A and B) normalized to β-actin (dashed line) 4 hours post treatment with IFN-γ. Data is represented as dCt, which is calculated by subtracting the Ct value of β-actin from the Ct of induced genes. All Ct values were acquired directly from qPCR with a maximum Ct threshold of 35. **(B)** Fold change in *CXCL10, CCL5, IL15, IL12A*, and *IFI16* expression in primary donor keratinocytes and matched-donor fibroblasts 4 hours post treatment with IFN-γ. Fold change calculated as relative to mock expression, normalized to β-actin, calculated using the ΔΔCt method. **(C)** IFNγ-induced gene expression at 4- and 24-hours post-treatment in keratinocytes and donor-matched fibroblasts (n = ≥3 donors with 3 replicates per donor). Symbols represent the geometric mean of each individual donor. ^*^P < 0.05, ^**^ *P* < 0.01, ^***^ *P* < 0.001, NS, not significant; linear mixed models were used to analyze fold change in gene expression.

Furthermore, we evaluated whether keratinocytes and fibroblasts exhibit differential gene expression in response to IFNγ incubation time by comparing gene induction at 4 hours and 24 hours post-treatment with 100 U/mL IFNγ. Keratinocytes exhibited significantly higher *CXCL10* induction than fibroblasts at both 4- and 24-hours post-IFNγ treatment (*P <* 0.001) (Fig 3C). Both cell types demonstrated increased *CCL5* expression at 24 hours post-treatment with no significant difference in induction between keratinocytes and fibroblasts (Fig 3C). While keratinocytes demonstrated higher induction of *IL15* and *IL12A* at 4 hours, fibroblasts achieved similar induction of *IL15* and more expression of *IL12A* compared to keratinocytes at 24 hours (Fig 3C). There was no difference in *IFI16* induction between cell types at 4 hours and 24 hours post-IFNγ treatment (Fig 3C). This dose-response analysis revealed that for *CXCL10*, *IL15*, *IL12A*, IFNγ elicits a faster and stronger antiviral gene response in keratinocytes compared to fibroblasts derived from the same donor. In addition, expression patterns for these cytokines vary with cell type and the timing of antiviral signaling.

### IFNγ induces significantly more *CXCL10* expression in asymptomatic individuals

We analyzed the difference in average gene induction in individuals who are asymptomatic or experience ulcerative HSV-2 reactivations to determine whether there is a correlation between HSV-2 disease severity and IFNγ-induced gene expression. Our analysis revealed that keratinocytes derived from asymptomatic individuals demonstrated higher *CXCL10* induction in response to IFNγ stimulation (Figs 4A and B). Although baseline *CXCL10* expression did not differ across donors, *CXCL10* expression in keratinocytes isolated from asymptomatic donors increased by 1,020-fold (33–6206) in response to the minimal dose of 0.1 U/mL IFNγ. Keratinocytes from persons defined as having ulcerative HSV-2 infection demonstrated a significantly lower average fold change in *CXCL10* induction, exhibiting only a 42-fold change (0.27-786) in *CXCL10* induction in response to the same dose of IFNγ (*P* < 0.01) (Fig 4A and B). Moreover, keratinocytes from asymptomatic persons consistently exhibited higher *CXCL10* induction at 1.0 and 10 U/mL IFNγ compared to individuals with ulcerative disease (*P* < 0.05) (Fig 4B). 10 U/mL IFNγ induced peak *CXCL10* expression in asymptomatic donors with an average 14,805-fold change in expression (Fig 4B). The equivalent dose was 5-fold less (mean 3,106-fold) in ulcerative donors (*P* < 0.01), with peak induction in donors with ulcerative disease reaching an average 6,294-fold following treatment with 100 U/mL IFNγ (Fig 4B). Other genes, including *CCL5, IL15, IL12A,* and *IFI16* did not exhibit significant differences in induction amongst donor cohorts; however, we consistently observed a non-significant trend of higher ISG expression in individuals with asymptomatic disease, particularly in response to minimal IFNγ stimulation (Fig 4B).

**Fig 4:**
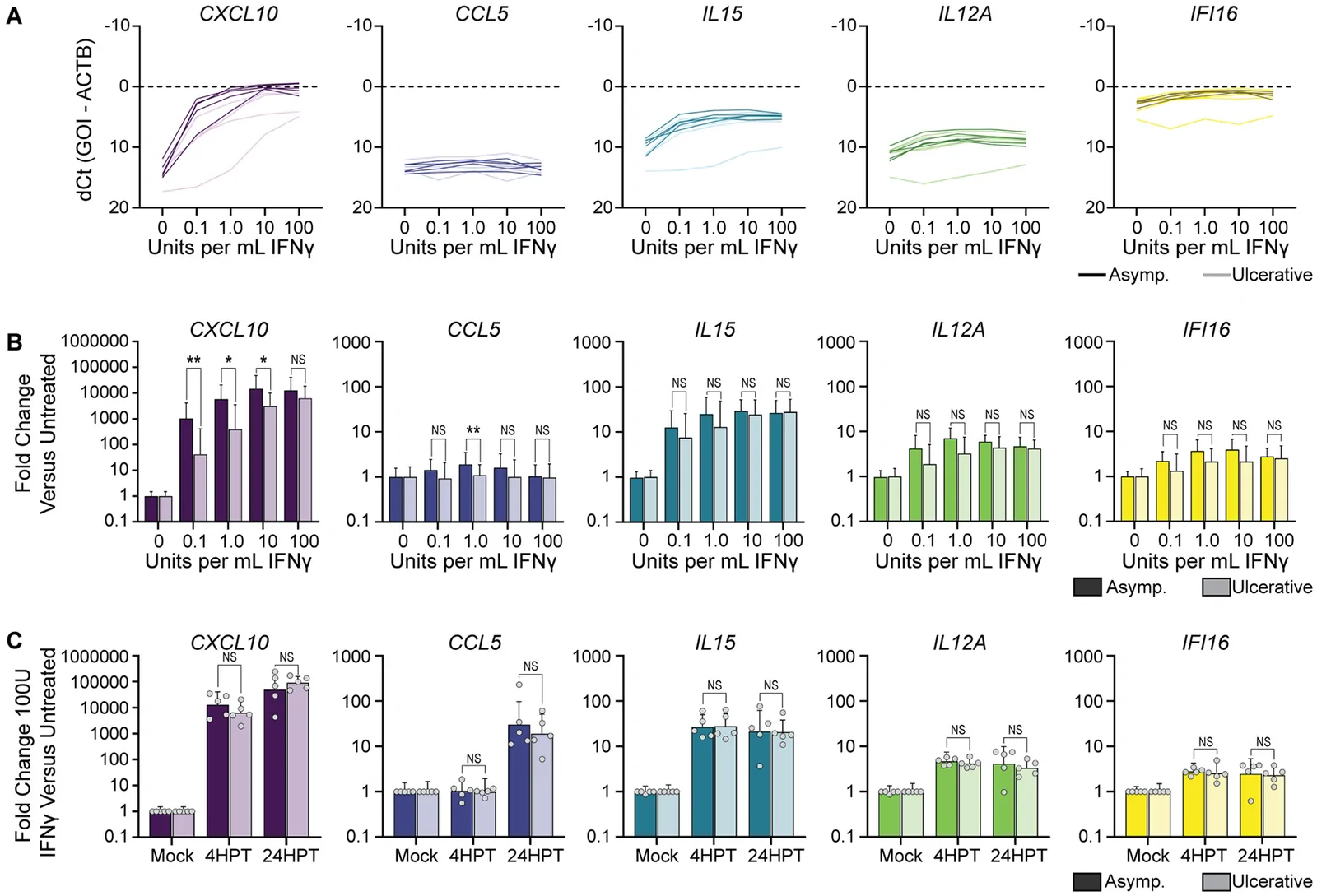
Comparison of Gene Induction in Response to IFNγ in Individuals with Asymptomatic Versus Ulcerative HSV-2 Disease. **(A)** Induction of *CXCL10, CCL5, IL15, IL12A*, and *IFI16* in response to different concentrations of IFNγ (0.1 to 100 U/mL) in primary donor keratinocytes derived from asymptomatic (colored solid lines) and ulcerative HSV-2 donors (light solid lines) 4 hours post treatment with IFN-γ. Each line represents the average of a single donor. Gene expression normalized to β-actin (dashed line). Data is represented as dCt, which is calculated by subtracting the Ct value of β-actin from the Ct of induced genes. All Ct values were acquired directly from qPCR with a maximum Ct threshold of 35. **(B)** Fold change in *CXCL10, CCL5, IL15, IL12A*, and *IFI16* expression calculated as relative to mock expression, normalized to β-actin, calculated using the ΔΔCt method 4 hours post treatment with IFN-γ. Darker bars represent asymptomatic cohort and lighter shade bars represent ulcerative cohort. **(C)** Fold change in *CXCL10, CCL5, IL15, IL12A*, and *IFI16* expression in asymptomatic (dark bars) and ulcerative cohorts at 4 and 24 hours post treatment with 100 U/mL IFNγ. Symbols represent the geometric mean of each individual donor. **(A-C)** ^*^P < 0.05, ^**^ *P* < 0.01, ^***^ *P* < 0.001, NS, not significant; linear mixed models were used to analyze fold change in gene expression. N = 5 donors with 3 replicates per donor, per cohort.

We observed no notable differences in gene induction between asymptomatic and persons with ulcerative disease following prolonged IFNγ stimulation (Fig 4C). Both cohorts demonstrated an increase in *CXCL10* and *CCL5* expression at 24 hours post-treatment, while the expression of *IL15, IL12A* and *IFI16* remained unchanged between 4 and 24 hours (Fig 4C). These findings suggest continuous transcription of chemotactic compounds that increase with time and controlled, maximum expression of other cytokines that remain stable for hours.

Finally, because we saw a delayed induction response in *CCL5* to IFNγ, we wanted to complete a dose response analysis at 24 hours post treatment to determine if there were any cohort differences in *CCL5* (S1 Fig). We observed that *CCL5* was not robustly induced by low doses of IFNγ even at 24 hours, requiring 10U/mL or more IFNγ to induce expression (S1A Fig). We observed a small but significant difference between our asymptomatic and ulcerative cohorts at 10U/mL but not 100U/mL (S1B Fig, P < 0.05).

### Comparative analysis of *CXCL9, CXCL10,* and *CXCL11* expression reveals robust chemokine expression in response to IFNγ

To determine whether IFNγ elicits induction of other CXC-chemokines, we compared gene expression of two related chemokines, *CXCL9* and *CXCL11* in donor-derived keratinocytes*.* CXCL9 has previously been shown to exhibit redundant properties with CXCL10*,* but some studies indicate unique temporal and spatial CXCL9 roles that differ from CXCL10 [58]. Data on CXCL11*,* especially in the context of HSV infection, is limited. After stimulating donor-derived keratinocytes with IFNγ, we observed robust induction of *CXCL9*, *CXCL10*, and *CXCL11* across all our donors (Fig 5A). All three chemokines were significantly induced following the lowest dose of IFNγ treatment (*P* < 0.001) and exhibited a strong dose-response curve with increasing IFNγ concentrations (Fig 5A). We observed that while *CXCL10* induction plateaued after 10 U/mL IFNγ, *CXCL9* and *CXCL11* expression continued to rise between 10 and 100 U/mL IFNγ (Fig 5A). Importantly, keratinocytes derived from individuals with asymptomatic disease consistently produced more *CXCL9* and *CXCL11* compared to their ulcerative counterparts, in agreement with our prior observations for *CXCL10* (Fig 5B).

**Fig 5:**
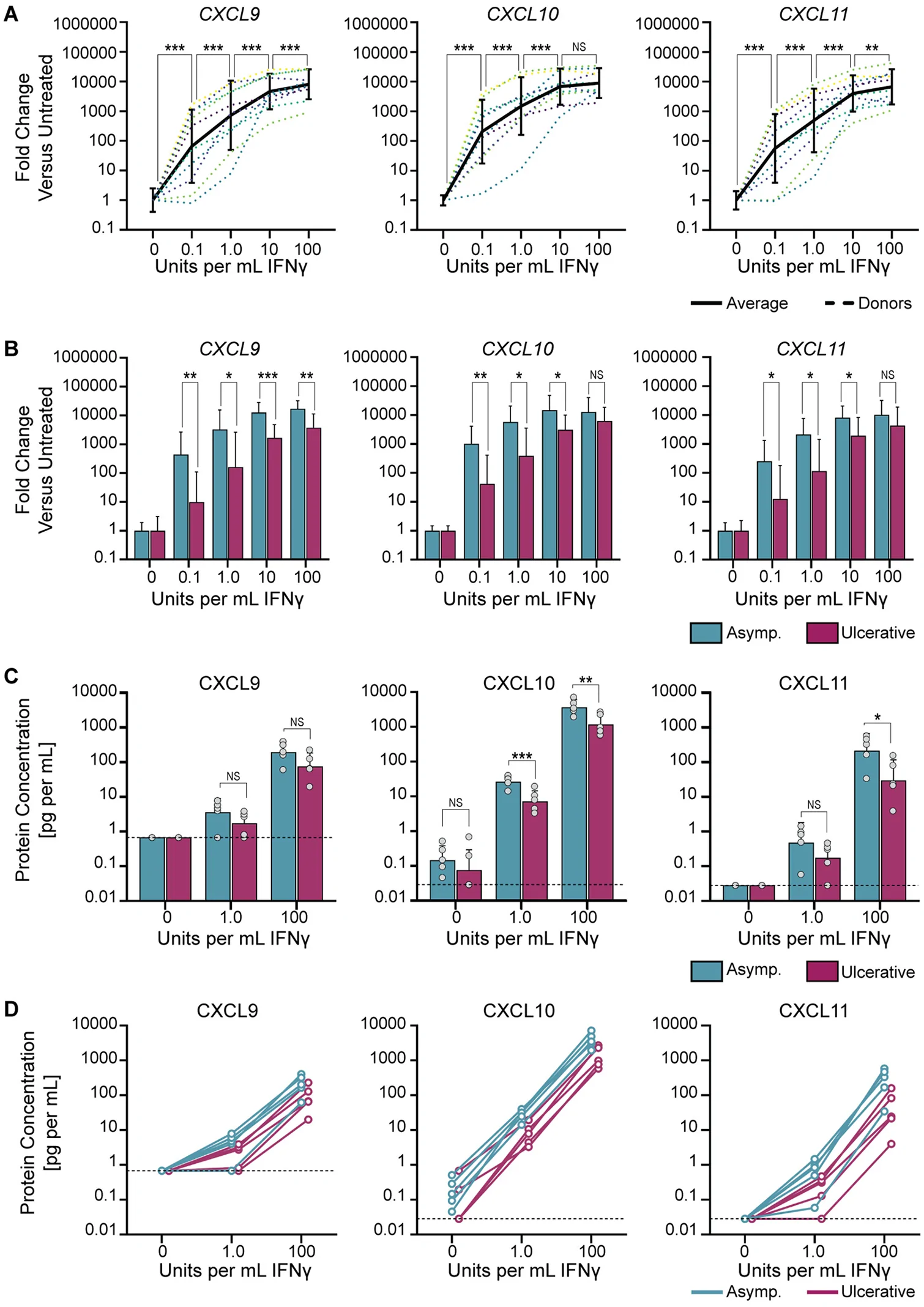
Comparison of CXC- Chemokine Expression in Response to IFNγ. Primary keratinocytes derived from individuals with asymptomatic or ulcerative HSV-2 disease were mock-treated or treated with increasing concentrations of IFNγ (0.1 to 100 U/mL) for 4 hours **(A** and **B)**. Log fold change in gene expression of *CXCL9, CXCL10,* and *CXCL11* was calculated relative to untreated group and normalized to β-actin. **(A)** Individual donor (colored dotted lines) and average donor gene induction (solid black line) of *CXCL9, CXCL10,* and *CXCL11* in response to IFNγ by primary donor keratinocytes. **(B)** Fold change in *CXCL9, CXCL10,* and *CXCL11* expression. **(C and D)** Cells were mock-treated or treated with 1.0 or 100 U/mL IFNγ for 12 hours, then CXCL9, CXCL10, and CXCL11 protein was detected in cell culture supernatant using Luminex assay. **(C)** Bars represent average mean protein per donor cohort; symbols represent means of individual donors. Dotted line for C and D represents a calculated limit of detection. **(D)** Each line represents an individual donor’s response to 0 U, 1 U and 100 U/mL IFNγ 12 hours post treatment **(A-D)** Blue bars represent asymptomatic cohort and magenta bars represent ulcerative cohort. ^*^P < 0.05, ^**^ *P* < 0.01, ^***^ *P* < 0.001, NS, not significant; linear mixed models were used to analyze fold change in gene expression. N = 5 donors, 3 replicates per donor, per cohort.

### Keratinocytes from asymptomatic individuals express elevated CXCL10 and CXCL11 protein

We next evaluated whether the RNA expression of highly induced CXC- chemokines translates to observable differences in protein production. Supernatant from keratinocytes mock-treated or treated with 1.0 or 100 U/mL IFNγ for 12 hours was collected and concentrated for a protein Luminex assay. IFNγ induced detectable CXCL9, CXCL10 and CXCL11 protein across all donors (Fig 5C and 5D). There was no significant difference in CXCL9 protein expression amongst the two cohorts at either IFNγ dose (Fig 5C). We detected significantly higher CXCL11 protein expression in asymptomatic donors, with over a 5-fold difference in CXCL11 expression following treatment with 100 U/mL IFNγ (*P* < 0.05) (Fig 5C). We observed higher CXCL10 protein expression in asymptomatic individuals following stimulation with both 1 U/mL and 100 U/mL IFNγ, with asymptomatic donors demonstrating over 3-fold difference in expression at 1.0 U/mL IFNγ and over a 2-fold difference following 100 U/mL IFNγ (*P* < 0.001, *P* < 0.01) (Fig 5C).

### Keratinocytes from asymptomatic donors exhibit increased pSTAT1 nuclear localization following IFNγ stimulation

To investigate the mechanism underlying the differential dose-dependent responses to IFNγ in keratinocytes from individuals with asymptomatic or ulcerative HSV-2 disease, we first assessed the overall antiviral efficacy of IFNγ. Keratinocytes were pretreated with various doses of IFNγ for 24 hours, and then infected with a recombinant GFP-expressing HSV-1 strain [59]. Live cell imaging and quantification of GFP fluorescence were used to measure inhibition of viral gene expression. We observed no significant difference in the inhibition of viral gene expression between keratinocytes isolated from asymptomatic donors and donors with ulcerative disease (Fig 6A and B), nor in the amount of IFNγ required to inhibit 50% of viral GFP expression at 24 hours post infection (*P* = 0.797) (Fig 6C). Furthermore, pretreatment with IFNγ resulted in similar inhibition of HSV-2 gene expression and replication in keratinocytes from both donor cohorts (S2 Fig). To determine whether differences in IFNγ signaling upstream of ISGs might explain differences in ISG expression, we compared baseline expression levels of the IFNγ receptor, IFNGR1, as well as the downstream mediator STAT1 in donor-derived keratinocytes and found no difference between asymptomatic and ulcerative donor pools (Fig 6D).

**Fig 6:**
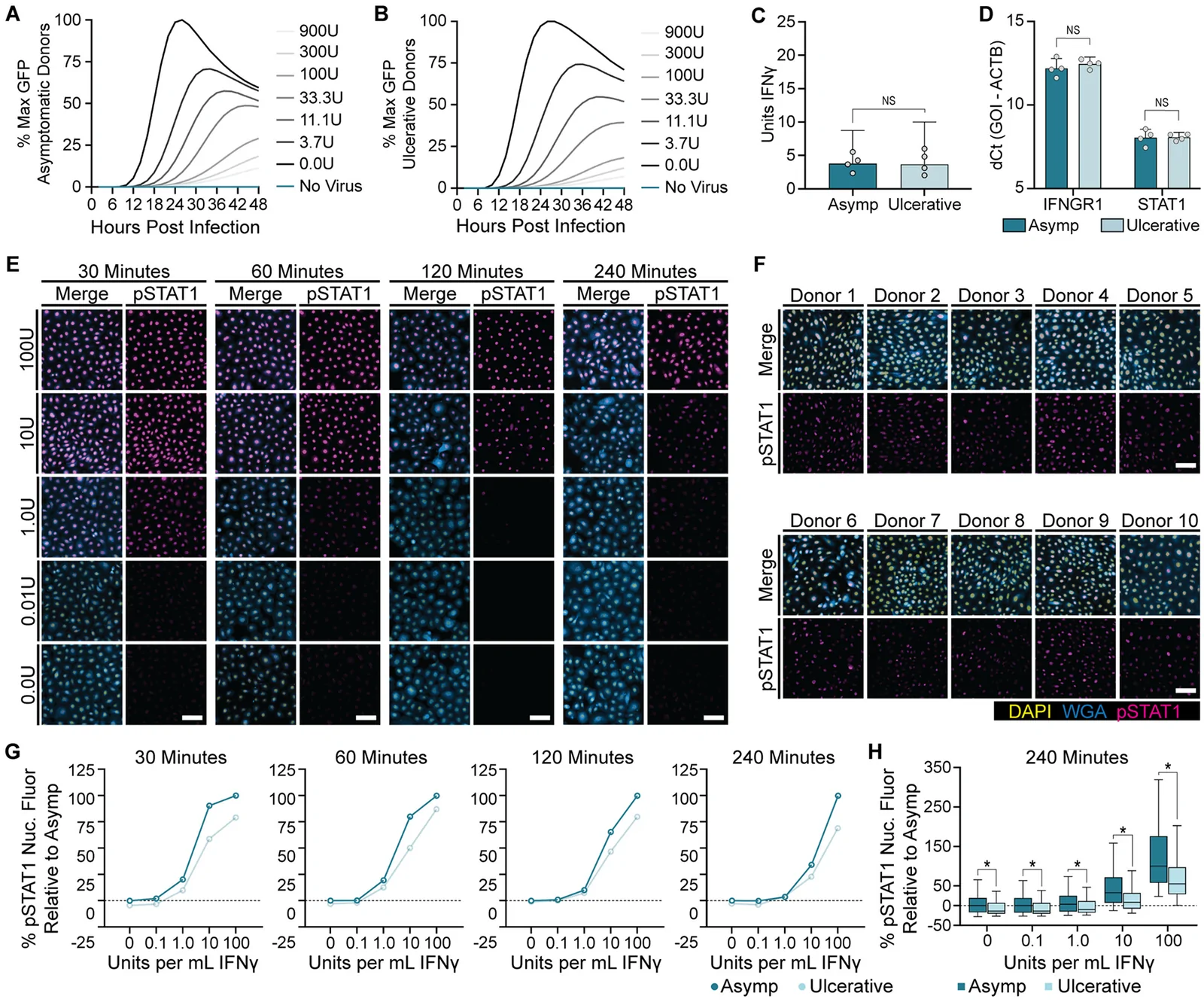
Upstream Variability in IFNγ Signaling Amongst Keratinocytes from Asymptomatic Donors and Donors with Ulcerative Disease. Keratinocytes isolated from asymptomatic donors **(A)** or donors with ulcerative disease **(B)** were pretreated with specified doses of IFNγ for 24 hours, then infected with GFP-expressing HSV-1. Live cell images were acquired every 2 hours for 48 hours and normalized to measure GFP fluorescence. **(C)** The amount of IFNγ required to inhibit 50% of viral GFP expression was calculated for keratinocytes from both asymptomatic and ulcerative donors at 24 hours post infection. **(D)** Expression of IFNGR1 and STAT1 was measured by RT-qPCR in untreated keratinocytes from asymptomatic or ulcerative donors. **(E)** Representative images from a single donor illustrating the detection of pSTAT1 in the nucleus of keratinocytes treated with various doses of IFNγ for the specified time. **(F)** Representative images from each donor at 60 minutes following treatment with 1U of IFNγ highlight heterogeneity in nuclear pSTAT1 signal. Yellow is nuclear DAPI staining, blue is membrane staining with wheat-germ agglutinin, and pink is pSTAT1. Scale bar represents 100 µm **(E** and **F)**. **(G)** Normalized average nuclear fluorescence of pSTAT1 at specified times following treatment with specified doses of IFNγ, separated by donor cohort. Data was normalized using the asymptomatic donor pool (0% set by 0U IFNγ, 100% set by 100U IFNγ). Each point is derived from the average fluorescence of at least 29,000 nuclei. **(H)** Box-and-whisker plot of raw nuclear fluorescence of pSTAT1 at 240 minutes following treatment with specified doses of IFNγ, separated by donor severity. Each bar represents the inner quartile range of raw fluorescence in at least 29,000 nuclei. ^*^P < 0.05, ^**^ *P* < 0.01, ^***^ *P* < 0.001, NS, not significant; Unpaired t-test models were used to analyze fold change in gene expression. N = 4 donors and 3 replicates per donor in each cohort **(A-D)** or 5 donors and 2 replicates per donor in each cohort **(E-H)**.

We next evaluated the level of nuclear pSTAT1 following treatment with multiple doses of IFNγ at 30-, 60-, 120-, and 240-minutes post treatment (Fig 6E) and identified a strong dose- and time-dependent response in nuclear localization of pSTAT1. There was a clear heterogeneous response at the cellular level, with some cells exhibiting bright nuclear staining of pSTAT1 while other cells demonstrated reduced staining. This heterogeneity was pronounced in all donors following treatment with 1.0 U/mL of IFNγ for 30 minutes (Fig 6F). Nuclear localization of pSTAT1 was dose-dependent, with elevated levels of pSTAT1 evident at all timepoints following treatment with 1.0 U IFNγ (Fig 6G). There was minimal nuclear localization of pSTAT1 detected following treatment with 0.1 U IFNγ at times greater than 30 minutes (Fig 6G). Pooling the response for all donors, keratinocytes from asymptomatic donors exhibited higher levels of pSTAT1 nuclear localization compared to ulcerative donor keratinocytes (P < 0.05) (Figs 6G and 6H). Together, these data indicate that keratinocytes isolated from asymptomatic and ulcerative donors develop resistance to viral infection following extended treatment with IFNγ and express similar levels of IFNGR1 and STAT1, but that there are differences in IFNγ signaling that manifest shortly after IFNγ binding that could drive downstream divergence in IFNγ signaling.

## Discussion

Our findings demonstrate that IFNγ elicits heterogeneous antiviral gene expression in primary human skin structural cells, revealing previously unappreciated donor- and cell type–specific variation. Across all donors, the closely related *CXCL9, CXCL10,* and *CXCL11* emerged as the most sensitive and robustly induced genes in response to IFNγ stimulation. *IL15* and *IL12A* also exhibited significant induction following treatment with IFNγ. Antiviral sensor *IFI16* was highly expressed in keratinocytes but only modestly induced by IFNγ. *CCL5*, another critical chemokine involved in antiviral immunity, was induced only at the later 24-hour timepoint post treatment with IFNγ. While both fibroblasts and keratinocytes exhibited robust induction of select antiviral genes, keratinocytes were more sensitive to IFNγ, demonstrating greater gene expression in response to IFNγ than fibroblasts. Furthermore, keratinocytes isolated from asymptomatic donors displayed markedly greater ISG induction at lower doses of IFNγ than those derived from donors with ulcerative disease, including more robust *CXCL9, CXCL10,* and *CXCL11* expression. Differential CXCL10 expression was evident at both the transcriptional and protein level, with three-fold greater protein production of CXCL10 detected in asymptomatic donors after 12 hours of treatment with a biologically relevant dose of IFNγ. An interrogation of upstream IFNγ signaling identified larger quantities of phosphorylated STAT1 in the nuclei of keratinocytes from asymptomatic donors versus ulcerative donors following IFNγ stimulation. Together, our analyses reveal several novel insights into IFNγ-mediated gene expression in primary cells derived from individuals with asymptomatic and ulcerative HSV-2 disease.

These patterns of response to IFNγ provide insights into the regulatory mechanisms of chemokine expression integral to viral containment during HSV-2 reactivation. CXCL10, a critical chemokine primarily responsible for T cell recruitment [44], is expressed rapidly and maintains elevated expression over time. In contrast, CCL5*,* another chemokine that recruits T cells, monocytes, and other immune cells, exhibits delayed expression post stimulation, suggesting it may play more of a role in immune maintenance and positioning rather than acute defense [60–62]. Prior work has demonstrated that herpes lesion vesicle fluid contains the β-chemokines CCL3, CCL4, and CCL5, and that HSV-infected keratinocytes can produce these chemokines *in vitro* although at a substantially lower level [63]. While our findings suggest that CXCL9, CXCL10, and CXCL11 likely mediate the early responses during HSV reactivation, they function within a broader chemokine network in which β-chemokines could coordinate cascade recruitment of lymphocytes, monocytes, and other immune cells as lesions evolve [64].

Although IL-15 and IL-12 are not primary mediators of chemotaxis, their rapid induction and sustained expression following IFNγ stimulation likely reflect roles in reinforcing local antiviral immunity through maintenance of T-cell effector function and amplification of IFNγ-dependent signaling pathways [47,49,51,53,54]. Interpretation of IL-12A induction warrants caution, as functional IL-12 depends on heterodimerization with IL-12B, which was not assessed in this study. Thus, increased IL-12A transcript alone should not be interpreted as evidence of increased functional IL-12 production. In contrast, IFI16 exhibited sustained expression consistent with its well-established role as a constitutively expressed sensor that contributes to intrinsic antiviral defense [41].

Comparison between donor-matched keratinocytes and fibroblasts suggests cell type–specific roles in IFNγ-mediated signaling. Keratinocytes exhibited stronger IFNγ–driven induction of *CXCL10*, *IL15,* and *IL12A,* positioning them as frontline effectors of antiviral defense that are acutely sensitive to early alarm signals such as IFNγ. Furthermore, the dramatic increase in *CXCL10* expression suggests that keratinocytes contribute to the formation of a chemotactic gradient, composed of chemokines such as CXCL10, that can influence the redistribution and retention [31,65] of local tissue resident memory T cells within the tissue and guide spatial positioning of circulating T cells to the site of infection. Human biopsy studies have indicated that prompt T-cell responses are associated with rapid containment during asymptomatic HSV reactivation [15,17]. Increased epidermal T-cell density, accumulation of TRM CD8 clusters, recruitment of circulating CD8 T cells, and elevated CD8 cytolytic functions have been documented in asymptomatic shedding episodes [17]. Our *in vitro* experiments appear to reinforce the anatomic understanding that T cells patrolling neurites in the skin would rapidly respond to infected keratinocytes following HSV reactivation, illustrated here by their exquisite sensitivity and responsiveness to IFNγ. Fibroblasts, in contrast, exhibited higher basal expression but weaker gene induction, suggesting complementary roles in immune cell population maintenance and regulation compatible with their presence in the upper to mid-dermis. Notably, both keratinocytes and fibroblasts displayed high constitutive *IFI16* expression, consistent with this sensor’s role in basal antiviral surveillance [41]. These findings support a division of labor in the stromal compartment: keratinocytes function as frontline sentinels that rapidly amplify chemokine/cytokine signals for immune recruitment and activation, while fibroblasts contribute to retention of tissue resident immune cell populations such as essential CD8 tissue resident memory T cells [66].

While there were consistent patterns of expression between ISGs, donors demonstrated broad variation in response to IFNγ. This donor-to-donor variation reflects meaningful biological diversity that is associated with the heterogeneous clinical outcomes observed in HSV-2 infection. For example, *CXCL10* demonstrated extreme variation in expression, ranging from an upregulation of 11.72-fold to 21,345-fold four hours post treatment with only 1.0 U/mL IFNγ. This variability in donor-specific responses was likewise observed at higher doses of IFNγ. Moreover, keratinocytes were more sensitive to IFNγ than previously described; a series of dose-response analyses involving a wide range of IFNγ concentrations revealed that peak gene induction occurred at less than or equal to 10 U/mL of IFNγ, which is much lower than quantities commonly used in previous *in vitro* studies, which range from 100U/mL to 1000U/mL [20,67–70]. At higher concentrations, donor-specific differences diminished, indicating that excessive IFNγ dosing may obscure biologically relevant variation in immune responsiveness. These findings argue for careful application of exogenous cytokines, including IFNγ, in experimental models to avoid artificial overexpression and to better capture regulatory dynamics with clinical relevance.

Heightened sensitivity to scarce IFNγ signals may be associated with a more immediate and effective antiviral state in asymptomatic individuals, potentially limiting the spread of reactivating virus before clinical disease manifests. CXCL10 has demonstrated efficacy as a vaccine adjuvant in mouse models [46], supporting the idea that elevated CXCL10 expression following IFNγ stimulation could contribute to improved clinical outcomes following viral reactivation. The sensitivity to IFNγ is particularly relevant for studying the response to HSV. Small populations of patrolling CD8 TRMs act as sentries that detect viral shedding from sensory nerve endings at the dermal-epidermal junction [15]. The production of IFNγ by tissue-resident memory T cells is essential for controlling HSV, but their limited numbers constrain the total amount of IFNγ available at sites of infection. Our finding that keratinocytes are highly sensitive to low doses of IFNγ suggests that a limited amount of IFNγ produced by immune cells *in vivo* is sufficient for structural cells to initiate a robust antiviral response.

The mechanisms underlying differential IFNγ responsiveness among donors remain unclear, but may involve epigenetic priming, genetic variation affecting JAK/STAT signaling, altered transcription factor activity, or crosstalk between IFNγ and other keratinocyte-derived cytokines, including type I interferons [71]. Notably, keratinocytes from asymptomatic donors exhibited modest but significant increases in nuclear pSTAT1 localization following IFNγ stimulation compared with keratinocytes from donors with ulcerative disease. Because signaling cascades can amplify small differences, enhanced STAT1 activation may contribute to the broad induction of IFNγ-stimulated gene observed in asymptomatic donors.

Despite these transcriptional differences, IFNγ-mediated inhibition of HSV replication was similar between groups. This may reflect the functions of the differentially induced genes, in particular, CXCL9, CXCL10, CXCL11, and CCL5, which primarily regulate immune cell recruitment and positioning rather than direct antiviral restriction. Differential CCL5 induction, unlike CXCL10, was only observed after prolonged stimulation and at higher IFNγ concentration. In contrast, induction of the antiviral effector IFI16 did not differ between cohorts. Together, these findings suggest that variation in IFNγ signaling preferentially influences inflammatory and chemotactic programs without substantially altering intrinsic antiviral responses. Enhanced chemokine induction in asymptomatic donors may therefore improve immune cell recruitment while maintaining comparable viral control. More broadly, these results support the possibility that naturally occurring variation in STAT1-regulated pathways contributes to heterogeneity in HSV disease severity, consistent with the broader importance of IFNγ signaling in viral disease outcomes, including SARS-CoV-2 [72,73].

We recognize that HSV reactivation does not occur in a stably recognizable pattern across all individuals—the variability in severity of outbreaks that occur within an individual indicates that host genetic factors are not the sole determinants of severity. However, variability in the timing and abundance of IFNγ expressed by TRM T cells may be a relevant factor in the pattern of disease expression. For *CXCL10*, which displayed the most dramatic difference in expression between asymptomatic donors and donors with ulcerative disease, underlying mechanisms including differential promoter activity, transcription factor affinity, or single nucleotide polymorphisms may explain observed expression disparities. Our finding that CXCL10 is upregulated in asymptomatic donors is further complicated by the role of CXCL10 as a proinflammatory cytokine. CXCL10 expression is necessary for successful viral clearance, but it can be deleterious in several viral, bacterial and autoimmune diseases where excessive inflammation cause significant pathologic effects [65,67,74,75]. In our analysis, we suspect that abundant CXCL10 production may be beneficial for resolving HSV symptoms [32,46]; however, functional assays with multiple cell types and a potential for spatial analysis would strengthen our hypothesis. Our findings suggest mechanistic insight into the wide clinical spectrum of HSV-2 infection and point toward host determinants that could serve as biomarkers or therapeutic targets.

This study is limited by the small number of participants and the subset of ISGs analyzed. Further studies are needed to expand our cytokine panel and translate these findings into more physiologically relevant models such as 3D organotypic models that will capture spatial diffusion of cytokines and cellular crosstalk. Furthermore, functional studies that modulate CXCL10 or upstream signaling will clarify causal roles in containment and provide a deeper understanding of structural cell–mediated immunity.

Collectively, our findings indicate that IFNγ responsiveness in structural cells varies between donors and cell types, and that these differences might be associated with HSV-2 disease severity. The ability of keratinocytes from asymptomatic individuals to mount strong ISG responses, including especially potent *CXCL10* induction at low IFNγ concentrations, may be associated with rapid, non-pathologic control of reactivation. We propose that differential ISG induction in structural cells may be associated with broad spectrum of clinical outcomes observed in HSV-2 infection. Elucidating the determinants of IFNγ sensitivity will be key to identifying host biomarkers of asymptomatic reactivation and optimizing therapeutic approaches that enhance mucosal antiviral immune responses against chronic viral infections.

## Materials and methods

### Ethics statement

The study protocol [STUDY00004312] was approved by the University of Washington Institutional Review Board Committee. Healthy, HSV-2 seropositive, and HIV seronegative adults were recruited by the University of Washington Virology Research Clinic in Seattle, Washington. Written informed consents were acquired from all participants. Participants were defined as having asymptomatic HSV-2 infection if they had no prior episodes of genital herpes or ulcerative HSV-2 infection with 6 or more recurrences per year.

### Primary cell isolation and culture

Three-millimeter punch biopsies were obtained from normal skin of the upper arm. Donor cells were isolated as previously described [76]. Biopsy samples were washed gently in cold PBS, then submerged in cold Dispase I solution (Sigma D4818) and incubated overnight at 4°C. The next day, biopsy samples were removed from Dispase I solution and placed into PBS. Epithelium was gently removed from the dermis using tweezers and placed into 0.6 mL tube containing 0.05% trypsin to dissociate keratinocytes (Gibco 25200–056). Epithelium samples were incubated at 37°C for 15 minutes, then mechanically dissociated using a blunt needle (Stemcell Technologies 28110) until the epithelial disc was visibly translucent. The trypsin solution was then neutralized with 5 mL of 10% FBS DMEM. Keratinocytes were collected and resuspended in 3mLs DermaCult Basal Medium (Stemcell Technologies 100–0501) supplemented with DermaCult expansion supplements (Stemcell Technologies 100–0502), 38µg hydrocortisone (Stemcell Technologies 07925), 125µg amphotericin B (Gibco 15290–019), 25mg streptomycin (Gibco 15140–122), and 25,000U penicillin (Gibco 15140–122) then seeded into collagen coated 6-well plates. Keratinocytes were treated with Rho kinase inhibitor Y-27632 dihydrochloride (Stemcell Technologies 72302) for the first 6 days of culture after isolation or until the first passage. Keratinocytes were passaged using trypsin 0.05% (Gibco 25200–056) and neutralized using 10% FBS DMEM supplemented with penicillin and streptomycin. All keratinocyte cells were used at passage 5 or less.

Separated dermis was placed into an empty well on a 6 well plate, cut into two portions and allowed to lightly air-dry to affix the tissue to the plate surface. 3 mL of CnT-Prime Fibroblast Proliferation Medium (Cell n Tec CnT-PR-F) was added to wells containing partially dry adhered dermis and all cell plates were incubated at 37°C. Fibroblasts were passaged using TripleE (Gibco 12604–013). Fibroblast cultures were used at passage 7 or less.

### IFNγ treatment

Interferon gamma (IFNγ, Thermo/PeproTech 300-02-100UG) was resuspended as a stock solution at 100,000 U/mL in PBS supplemented with 0.1% bovine serum albumin (Sigma B4287) and stored at -20°C. Primary human keratinocytes were seeded into 12-well plates at 100,000 cells per well, then allowed to incubate overnight. The next morning, IFNγ was serially diluted in media to generate working solutions of 0.1, 1.0, 10, or 100 U/mL. Keratinocytes were left untreated as a control or treated with specified amounts of IFNγ for specified periods of time.

### Live cell imaging

96-well plates (Revvity 6005810) were collagen coated, seeded with 5,000 keratinocytes per well, which were allowed to attach overnight before being treated with specified doses of IFNγ and incubated for an additional 24 hours at 37°C. Following incubation with IFNγ cell media was changed and cells were infected with HSV-1 K26 [59] or HSV-2 HG52 del47 [77]. Live cell imaging was collected with Sartorius Incucyte SX3 to measure GFP fluorescence every two hours. Live cell images were analyzed by Incucyte software version 2020c to determine integrated GFP fluorescence intensity (GFP fluorescence x image area). To normalize GFP fluorescence, the maximum integrated intensity value for infected samples not treated with IFN-γ was set to 100%, while the minimum integrated intensity value in uninfected samples was set as 0%. The dose-response inhibition of viral GFP expression was calculated by four-parameter multiple linear regression of GFP integrated intensity values at 24 hours using GraphPad Prism version 10.2.1.

### RNA extraction and cDNA synthesis

Following treatment with IFNγ or infection with HSV, cells were directly lysed in their wells using QIAGEN RNeasy (QIAGEN 74104) or Zymo Research Quick-RNA MiniPrep (R1054). Lysate in RNeasy kits was homogenized using QiaShredder Mini Spin Columns (QIAGEN 79654). Samples were processed through RNeasy or Quick-RNA kits per manufacturer’s protocol. Purified RNA was stored at -80C or immediately synthesized into cDNA following SuperScript IV First-Strand Synthesis protocol (Thermo/Invitrogen 18091050). cDNA was stored at -20°C.

### RT-PCR

cDNA amplification was performed using TaqMan Universal Mastermix II (Thermo/Life Technologies 4440048), one microliter of cDNA, and premade commercial TaqMan probes for *CXCL10*, *CCL5*, *IL15*, *IL12A*, *IFI16*, and beta-actin (Thermo). Samples were run in technical duplicates. The average Ct value between sample technical replicates for each probe was used to calculate ∆Ct and ∆∆Ct with a maximum Ct threshold 35. Fold change in gene expression was calculated relative to mock expression levels and with respect to beta-actin, the housekeeping gene used in this study.

### DNA PCR for viral titers

A 96-well plate (Revvity 6005810) was collagen coated, then keratinocytes were seeded at 5,000 cells per well and allowed to attach overnight before being treated with IFNγ. 24 hours later cells were infected with HSV-2 186 at an MOI of 1. Infected cells and supernatant were collected 24 hours post infection and DNA was extracted using the Zymo Research Quick-DNA 96 Plus kit (Zymo Research D4070) following the manufacturers protocol. Total viral genomes were detected using PCR and quantified by standard curve. Standard curve stock material is previously-extracted DNA from culture isolates, ddPCR-quantified, and serially diluted. All qPCR reactions were a total volume of 30uL, including 4.495μL water, 14.333μL No-ROX QuantiTect master mix (Qiagen), 0.667μL ROX QuantiTect master mix (Qiagen), 0.030μL of Uracil-N-Glycosylase (0.025 units, EpiCentre technologies), 8e4 copies of EXO (internal control) template DNA, and 10μL of template DNA. Primer and probe concentrations were 1600nM each of assay-specific forward (gB CGC ATC AAG ACC ACC TCC TC) and reverse (gB GCT CGC ACC ACG CGA) primers, 150nM gBTyp1 probe (VIC-TGG CAA CGC GGC CCA AC-QSY), 100nM gBTyp2 probe (FAM-CGG CGA TGC GCC CCA G-QSY), 55.6nM EXO primers, 83.8nM EXO probe. Amplification on the QuantStudio 7 Flex (ThermoFisher) consisted of 2 minutes at 50C and 15 minutes at 95C, followed by 45 cycles of 1 minute at 94C and 1 minute at 60C with acquisition, with all ramp rates set to 1.6C/s.

### Protein analysis

Luminex assay was performed by the Fred Hutch Cancer Center immune monitoring core to measure protein levels of CXCL9, CXCL10 and CXCL11 in cell culture supernatant. 12 well plates were collagen coated, seeded with 100,000 keratinocytes per well, then allowed to incubate overnight at 37°C. The next day, cells were mock-treated or treated with 1U or 100 U/ML of IFNγ in triplicate. Supernatant was collected at 12 hours post treatment with IFNγ, triplicates were pooled together, and the resulting supernatant mixture was concentrated using 3kDa cutoff spin filters (Sartorius VSO4T92). Concentration factors were determined by dividing the input supernatant volume by the total volume collected after concentration. Protein levels determined by Luminex in concentrated samples were divided by each sample’s unique concentration factor to determine the total amount of protein present in the original unconcentrated supernatant. The limit of detection in concentrated samples was determined by dividing the assay’s original limit of detection by the average concentration factor across all samples.

### Immunofluorescence staining

Primary human keratinocytes were cultured in DermaCult Basal Medium (Stemcell Technologies 100–0501) and seeded at 10,000 cells/well in collagen coated 96-well plates (Revvity 6005810). Cells were incubated at 37°C overnight, then mock-treated or treated with 0.1, 1.0, 10, and 100 U/mL IFNγ for 30, 60, 120, or 240 minutes. Cells were fixed in 4% formaldehyde (Boster Bio AR1068) in PBS for 15 minutes at room temperature. Cells were permeabilized by incubation in 100% methanol at -20°C for 10 minutes. Samples were blocked using a blocking buffer (0.3% Triton X-100, Acros 327371000) and normal donkey serum (Jackson Labs 017-000-121) in PBS. Primary Tyr701pSTAT1 (1:800 Cell Signaling Technology) antibody was diluted in antibody buffer (0.3% Triton X-100 and 1% BSA in PBS) and incubated overnight at 4°C. The next day, cells were rinsed in PBS and incubated with AlexaFluor647 conjugated secondary antibody (Invitrogen) diluted at 1:200 in antibody buffer for 2 hours at room temperature. Cells were then counterstained using AlexaFluor488-conjugated wheat germ agglutinin (Thermo W11261) for 15 minutes at room temperature, washed, mounted using SlowFADE Diamond plus DAPI (Thermo 536964), and imaged using Nikon Eclipse Ti2. All staining and imaging were completed on the same day using the same microscope acquisition settings. All image analysis was completed using the Nikon Elements software suite version 5.21.03.

### CellProfiler analysis

All images were processed using the same CellProfiler (version 4.2.8) pipeline, which corrected illumination using a median filter and 125 smoothing filter size to mitigate imaging artifacts. DAPI was used to identify nuclei as primary objects; primary object identification used global minimum cross-entropy thresholding with a specified size of objects capped at 60 pixels. Primary object shape and intensity was used to separate clumped nuclei. Overlay images were generated for quality control. pSTAT1 fluorescence was measured within the DAPI identified nuclei primary objects. Mean pSTAT1 fluorescence was calculated by CellProfiler for each nucleus, then exported for graphing in GraphPad Prism.

### Statistical analysis

Figures were generated using GraphPad Prism version 10.5.0. Statistical analyses were performed using R 4.5. Expression data were analyzed on the log10 scale to compensate for the multi-log range of upregulation seen in CXCL9, CXCL10, and CXCL11 expression. Fluorescence data were analyzed by computing the median of the integrated intensity values across nuclei within each sample. Linear mixed models were used to analyze data to account for the random effects of donor variability. Alternatively, unpaired and paired t-tests are included within the statistical analysis report. A *p* value of < 0.05 was considered significant. Statistical analysis and raw data used for figure generation can be found in the GitHub repository. Unpaired and paired t-tests are included within the statistical analysis report: https://github.com/youyifong/donor-keratinocyte-fibroblast-ifng.

## Supporting information

S1 Fig Comparison of CCL5 Induction in Response to IFNγ at 24 hours post treatment in Asymptomatic Versus Ulcerative HSV-2 Disease. (A) Fold-change of CCL5 expression in response to IFNγ at 24 hours post treatment (N = 8 donors, 3 biological replicates per donor). CCL5 expression was calculated as relative to mock expression, normalized to β-actin, calculated using the ΔΔCt method. (B) Comparison of fold-change in CCL5 expression in keratinocytes from asymptomatic or ulcerative donors (N = 4 donors, 3 biological replicates per cohort). * P < 0.05, linear mixed model.
(TIF)

S2 Fig Pretreatment with IFNγ inhibits viral gene expression and replication in keratinocytes from asymptomatic donors and donors with ulcerative disease. Keratinocytes were pretreated with specified doses of IFNγ for 24 hours, then infected with either HSV-1 K26 (A), HSV-2 HG52 del47 (B), or HSV-2 186 (C). Gene expression was quantified in (A) and (B) using the Incucyte live cell imagining platform to capture expression of virus-encoded GFP. Total viral genomes were calculated by qPCR from the combined supernatant and cell lysates for HSV-2 186 (C). All data were collected at 24 hours post infection from n = 4 donors per cohort and 3 biological replicates per donor.
(TIF)

S3 Data Raw data used to generate Figs 1–6 and S1–S2 Figs. Each figure has its own worksheet. Each donor has three columns, one per biological replicate. Where applicable, each sheet also includes the statistical analysis output that goes with the figure. Raw data for Fig 6G and 6H are too large to include here and are available at https://github.com/youyifong/donor-keratinocyte-fibroblast-ifng.
(XLSX)
